# *In vitro* study of the effect of resveratrol purified from the skin of Iraqi black grape (*Vitis vinifera*) on lymphocyte cultures isolated from the blood of patients with lymphoma

**DOI:** 10.25122/jml-2022-0038

**Published:** 2022-06

**Authors:** Zainab Yaseen Mohammed Hasan, Fatma Abd Alhamza Obed, Ahmed Abdulmunem Jasim, Alaa Fadhil Alwan

**Affiliations:** 1 Department of Health Science, Biotechnology Research Center, Al-Nahrain University, Baghdad, Iraq; 2 National Center of Hematology, Mustansiriyah University, Baghdad, Iraq

**Keywords:** resveratrol, lymphoma, non-Hodgkin lymphoma, Hodgkin lymphoma, TNF-α, IL-10

## Abstract

The natural stilbene compound resveratrol (RSV) was extracted and purified locally from the black grape skin (*Vitis vinifera*) cultivated in Iraq. Cultures of human peripheral lymphocytes were obtained from the blood samples of patients with and without lymphoma to be treated with RSV at different concentrations. Three RSV concentration levels were subjected to isolated lymphocytes from blood samples of Hodgkin lymphoma (HL), non-Hodgkin lymphoma (NHL), and without lymphoma to estimate the change in TNF-α and IL-10. Resveratrol seemed to differently affect cytokines level in normal and lymphoma lymphocytes in relation to its concentration. The lowest resveratrol concentration (50 µg/ml) decreased TNF-α levels for patients without lymphoma and all NHL patients in contrast to the HL sample. Treating normal lymphocytes with a higher dose (1000 µg/ml) might elevate the levels of TNF-α in almost all samples. There was an inverse relationship between both cytokines in most treatments; with the increase in TNF-α level, there was a decrease in IL-10 level except in HL and normal lymphocytes treatment. The locally purified resveratrol could serve as a multi-target drug that modulates the immune system to improve body defense in patients suffering from lymphoma and in patients without lymphoma by altering cytokine levels in response to different resveratrol concentrations in a different manner.

## INTRODUCTION

The search for novel and effective cancer chemo-preventive agents led to the identification of various naturally occurring phytochemical compounds, one of which is resveratrol (3,5,4'-trihydroxy-trans-stilbene), a stilbenoid, a type of natural phenol, and a phytoalexin produced naturally by several plants in response to injury or when the plant is under attack by pathogens such as bacteria or fungi [1]. Food sources of resveratrol include the skin of grapes, blueberries, raspberries, and mulberries [2]. Resveratrol has potent anti-inflammatory, antioxidant, anti-platelet aggregation effects, and cardiovascular protection [3–5]. In addition, many studies found that resveratrol can affect cancer initiation, promotion, and progression, which raised hopes that it has potential for prevention and treatment [6].

Resveratrol is a new safe drug for the treatment of many diseases, especially cardiovascular disorders, as authorized by the World Health Organization (WHO) and the Food and Drug Administration (FDA) [7, 8]. Iraqi black grapes are rich with resveratrol [2], and a few studies were done about its human benefits in the country. Therefore, we used the pure resveratrol extracted from the locally grown black grapes registered as a novel certificate for industrial property according to certificate number (1642) on 18/5/2016/Baghdad/IRAQ. This study assessed the effect of the locally purified resveratrol (RSV) on cultured human blood lymphocytes suffering from lymphoma by estimating cell viability using an MTT assay of the treated cells with different concentrations of the extracted resveratrol compared to untreated control cells.

Furthermore, we evaluated the cellular (interleukins) levels after exposing these isolated lymphocyte cultures for different periods compared to normal cytokine levels in the lymphocyte culture of patients without lymphoma. This study aimed to assess the effect of RSV natural substances on the lymphocytes of lymphoma (NHL&HL) patients.

## MATERIAL AND METHODS

### Plant collection, resveratrol extraction and purification

All resveratrol extraction and purification processes were mentioned in the previous studies [9, 10]. Briefly, the purified resveratrol was obtained after extraction of fresh grape skin with 80% v/v ethanol, hydrolysis with 10% HCl solution, and the aglycon moiety was taken with chloroform. The preparative thin-layer chromatography (PTLC) technique was used for purification using silica gel G60 packed glass plates with mobile phase benzene: methanol: acetic acid 20:4:1 to obtain pure crystals identified as resveratrol (mixture of two isomers cis and trans) in relation to resveratrol standard, and about 35 mg resveratrol crystals/0.5 kg fresh grape skin was obtained as a result of these processes.

### Lymphocyte culture and viable counting

Lymphoma outpatients at National Center of Hematology/Mustansiriyah University, Baghdad/Iraq, with an age range of 35–60 years, and a healthy volunteer; were included in this study after collecting five milliliters of peripheral venous blood [11]. The blood of each patient was transferred into vacuumed heparinized tubes with gentle rolling. A general protocol for lymphocyte separation [12] was performed with the separating solution (Human lymphprep; sp. g=1.077 g/l) to collect lymphocytes pellets by centrifuging samples at 2000 rpm for half an hour. The collected lymphocyte cells were suspended in a 10 ml complete growth medium after washing several times with RPMI medium and centrifuging for 10 min at 2000 rpm. Counting living lymphocytes was done with trypan blue solution dye and the haemato-cytometer chamber as routine work.

### Determining the effect of the purified resveratrol on lymphocytes proliferation using MTT assay

The suspended isolated lymphocyte cells of each sample were subjected to different sterilized concentrations of purified resveratrol, including (7.5, 3.75,1.875, 1.0, 0.5, 0.25, 0.12, 0.06, 0.03, and 0.015) mg/ml using the 96 well microplate, incubated for 20 h at 37℃ in a CO_2_ incubator [12]. The negative control was represented by untreated lymphocyte cells from each patient and the healthy person and suspended in the medium. The MTT dye (2 mg/ml) was added to all wells and incubated for 4 h to be metabolized by living cells. Finally, dimethyl sulfoxide (DMSO) solution was added to dissolve the formed purple crystals and read at 620 nm with an ELISA reader. The percentage of lymphocytes viability was calculated as follows:

[*Absorbance of the test I negative control*]× 100

The three selected extracted resveratrol concentrations that may cause lymphocyte proliferation were chosen to study their effects on different interleukin levels.

### Determining the effect of purified resveratrol on cytokines levels using the ELISA technique

The assessment of cytokine (IL10 and TNFα) levels in lymphoma patients before and after exposure to three selected resveratrol concentrations was employed to compare the levels of interleukins secreted by lymphocytes subjected to purified resveratrol with normal levels for 3 hours of drug exposure.

### Sample collection

Seven patients with a diagnosis of non-Hodgkin lymphoma relapse were enrolled in this study. Blood samples were collected for patients under periodic care in the National Center of Hematology Baghdad/Iraq. Information about gender, age, history of patients, and all treatments was collected.

### Lymphocytes isolation and culturing

The general method for lymphocyte isolation and culturing was employed as mentioned in the previous section; each isolated patient lymphocyte was re-suspended in a 3 ml RPMI 1640 medium [12].

### Work Procedure

- 500 µl isolated lymphocyte suspended cells were seeded in each 24 well tissue culture plate (1X104 cell/well). Each patient had their own control, and all treatments were applied in duplicate.
- All plates were incubated for at least 2 h in a CO_2_ incubator before treatments so that cells could rest after isolation.
- Three selected resveratrol concentrations were chosen: 1000, 250, and 50 µg/ml, given the different ranges in proliferative effect for the lymphocytes in the previous work mentioned above. All solutions were sterilized with a 0.22 µm Millipore filter.
- Each resveratrol concentration aliquot of 500 ml was added to each well in duplicate. Negative control was represented by untreated patient cells suspended in a growth medium, and then the plates were incubated for 3 h in a CO_2_ incubator at 37℃.
- At the end of interval times, all wells were aspirated and transferred in separated vacuum tubes labeled for each patient and centrifuged for 20 min at 2000 rpm.
- The supernatants of each tube were separated and kept at -20℃ to be estimated by ELISA kit.

### Cytokine IL-10 and TNF-α levels

The supernatants of the treated lymphocytes with purified resveratrol were subjected to the kit protocol, following the instruction of the "US Biological TNF-α and IL10" kit protocol with Catalog No T9160-01. The level of the studied interleukins in each sample was measured according to the straight-line equation of the plotted standard curve for each cytokine concentration against their absorbance at 450 nm.

## RESULTS

Content and all purification analysis results of resveratrol from Iraqi *Vitis vinifera* skin fruits were mentioned in a published paper [9].

### Effect of the purified resveratrol on lymphocytes proliferation by MTT assay

**Figure 1:**
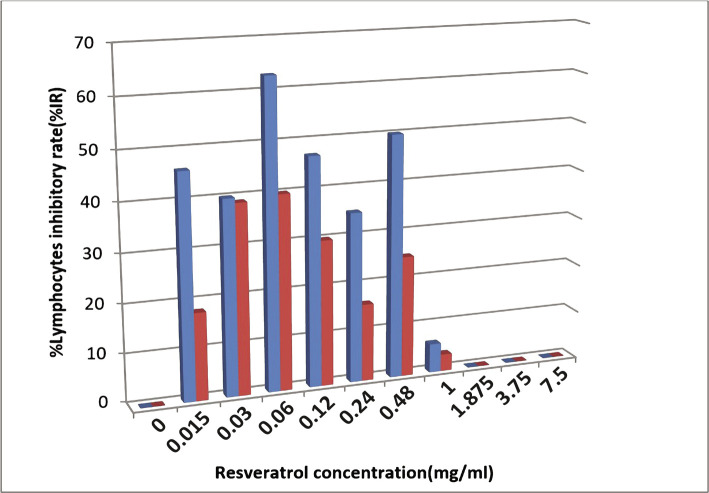
%IR results for ten resveratrol concentrations that affected normal human lymphocytes (red) and lymphoma lymphocytes (blue) by MTT assay after 24 hours of exposure.

Figure 1 showed MTT assay results for ten resveratrol concentrations that affected normal human lymphocytes (red bar) and lymphoma lymphocytes (blue bar) after 24 h exposure.

The first highest resveratrol concentration (1 mg/ml) that affected the lymphoma lymphocytes (%IR=6) with less cytotoxic effects on normal inhibition rate (%IR=3.5) was chosen for cytokines detection. Resveratrol with a concentration of 0.24 mg/ml showed lymphoma lymphocytes inhibition rate (%IR=35) while causing normal lymphocytes inhibition rate (%IR=16). The lowest resveratrol concentration of 0.015 mg/ml inhibited normal lymphocytes (%IR=18), and the lymphoma lymphocyte inhibition rate reached %IR=46. Thus, we identified the three best resveratrol concentrations to evaluate interleukin levels in lymphoma with the lowest cytotoxic effect on normal lymphocytes (1000, 250, and 50 µg/ml).

### Effect of resveratrol on cytokine levels (IL-10 and TNF-α)

**Figure 2:**
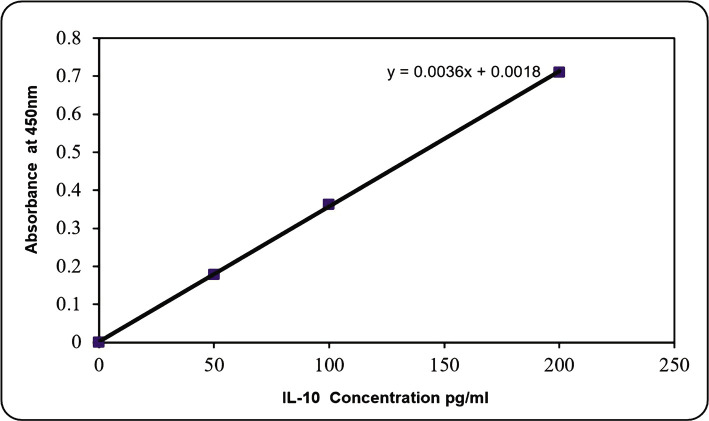
Interleukin-10 Standard Curve.

**Figure 3:**
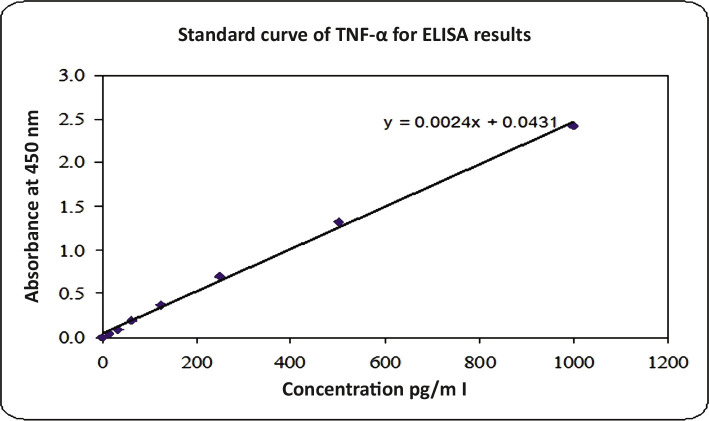
TNF-α Standard Curve.

In order to trace cytokines (IL-10 and TNF-α) levels in the supernatant of the treated lymphocytes with resveratrol at three selected concentrations for 3 h exposure on each patient as well as normal lymphocytes with their controls, the ELISA technique was used, and standard curve for both interleukins was plotted separately (Figures 2 and 3).

Table 1 shows the number, gender, age, and diagnosis status of the blood samples of patients used for analysis.

**Table 1:** Descriptive characteristics of participants.

Patient number	Gender	Age (years)	NHL/HL	Comments
**1**	Male	57	NHL	3 years with medications
**2**	Female	75	NHL	2 years with medications
**3**	Male	53	NHL	3 months with medications
**4**	Female	65	NHL	7 months with medications
**5**	Female	65	NHL	8 months with medications
**6**	Female	32	NHL	5 years with medications
**7**	Male	16	HL	8 months with medications
**8**	Male	35	Normal person	No medication along the study period

HL – Hodgkin lymphoma; NHL – non-Hodgkin lymphoma.

The concentration of cytokines for all samples was calculated with and without resveratrol treatment (Tables 2 and 3), according to the straight-line equation applied in Figures 2 and 3.

**Table 2:** Levels of IL-10 for all samples with and without resveratrol treatments in three concentrations for 3 h intervals.

Patient Number	IL-10 level (pg/ml) after three hours of resveratrol (mg/ml) treatment				LSD value
	0	0.05	0.25	1.0	
**1**	27	26.7	25.7	26	2.98 NS
**2**	28	28.7	26.7	26.7	2.66 NS
**3**	27	31	28.7	27.7	3.25*
**4**	30.33	31.33	29	30.33	2.84 NS
**5**	28	29.7	27.7	29	2.73 NS
**6**	41.33	28.7	29	28.33	4.38*
**7**	33.7	37.7	43.7	32.7	4.07*
**8 (control)**	26.3	25.7	27	27.7	2.36 NS

* – P≤0.05; LSD – least significant difference.

**Table 3:** Levels of TNF-α for all samples with and without resveratrol treatments in three concentrations for 3 hours intervals.

Patient Number	TNF-α level (pg/ml) after three hours of resveratrol (mg/ml) treatment				LSD value
	0	0.05	0.25	1.0	
**1**	363	237	238	397	52.44*
**2**	463	190	193	574	72.19*
**3**	446	191	229	227	70.94*
**4**	326	230	252	497	61.22*
**5**	374	262	180	599	85.92*
**6**	380	297	226	383	72.11*
**7**	229	244	286	343	64.02*
**8 (control)**	257	188	235	313	58.63*

* – P≤0.05; LSD – least significant difference.

The levels of IL-10 and TNF-α for the patient without lymphoma (26.3, 257 pg/ml) were lower than those of all samples involved in the study (Tables 2 and 3). Resveratrol seemed to differently affect the cytokine levels in normal and lymphoma lymphocytes in relation to its concentration. The lowest resveratrol concentration (50 µg/ml) decreased TNF-α level for the patient without lymphoma and all NHL patients in contrast to HL sample, while there was little increase and decrease or no effects in the case of IL-10 level in all samples. Furthermore, resveratrol at the concentration of 250 µg/ml had the same effects on TNF-α and IL-10 levels as the previous dose. Treating normal lymphocytes with resveratrol (1000 µg/ml) might elevate TNF-α levels in almost all samples and generate an inverse relationship between both cytokines when using this treatment. As the TNF-α level increased, there was a decrease in IL-10 level except in HL and normal lymphocytes treatment.

## DISCUSSION

Recently, more and more studies have focused on the different effects of this natural plant, stilbene resveratrol (RSV), as a promise anti-carcinogenic, anti-tumor, anti-inflammatory drug, besides its role in enhancing the sensitization of radiotherapies and chemotherapies [13]. These effects triggered *in vitro* and in vivo research for lymphoma and leukemia cells at the cellular and molecular targeting level. RSV might possess several pathways involved in these responses [14].

Two distinct functions of cytokines promoted by T lymphocytes were generated: the first was T helper one (Th1) cytokines mediated by IL-2 and IFN-c and acted to initiate the cell-mediated immunity that played a role in tumor destruction. For T helper two (Th2) response, the cytokines involved were IL-4, IL-10, and IL-13 which act to inhibit the cell-mediated immune response as a rule in the negative regulation of tumor immune surveillance, which diminishes nitric oxide production by monocytes and macrophages that suppressed apoptosis and CTL response [15].

Thus, we studied the expression of TNF-α and IL-10 as Th1 and Th2 mediated cytokines, respectively, involved in lymphoma patients in Iraq, especially given the high incidence of blood disease in this country [16, 17]. Moreover, we aimed to detect the effect of purified resveratrol treatments on lymphoma patients in comparison to lymphocytes *in vitro*. The obvious results indicated that a high resveratrol dose (1 mg/ml) affected Th1 and Th2 mediated cytokines by increasing both TNF-α and IL-10 levels in reverse to the RSV low doses for normal human lymphocytes. For lymphoma patients with NHL and HL, TNF-α and IL-10 levels were higher than normal levels before RSV treatment. In lymphoma isolated cells treated with RSV, irrespective of doses, whenever there was a decrease in one cytokine (*e.g*., TNF-α), there was an increase in the level of the other (*e.g*., IL-10) and *vice versa*.

The immune-modulation expressed by resveratrol might deal with its inhibitory action upon the transcriptional factor NF-kappaβ that led to decreased TNF-α, which is linked to regulation of cell proliferation and apoptosis as immune responses. Resveratrol could be considered a promising chemotherapeutic agent involved in anti-cancer mechanisms, and the current results agreed with the suggestions of other authors [18]. Resveratrol in different doses resulted in different effects. In a study by Raffaele et al. [19], it was concluded that treatment with low doses of RSV might cause cell cycle arrest at S-phase in Hodgkin lymphoma (HL) patients. Higher RSV doses may initiate caspase-3 in lymphoma cells leading to apoptosis [19].

Resveratrol induced NKTCL cells apoptosis through mitochondrial pathway in cases of extra-nodal NK/T cell lymphoma (NKTCL) that are commonly non-Hodgkin lymphoma affecting Asians and Central and South Americans [20].

Other authors emphasized that RSV may act as a down-regulation of the MCl-1 gene with up-regulation of Bex and Bad, which led to activation of caspase-9 and caspase-3 [21].

In addition, researchers found that RSV directly activated the DNA damage response (DDR) pathway by shutting down AKT and Stat3 phosphorylation in a study for etoposide and ionizing radiation [22]. The up-regulation of Zita of Epstein-Barr virus (EBV) caused by administration of RSV in lymphoma patients could result in cell proliferation inhibition and apoptosis of abnormal cells [23].

Some authors suggested that RSV might play a unique role in the differentiation of anaplastic large cell lymphoma (ALCL) cells representing a specific mature B-cell neoplasm bearing CD30 surface marker. Thus, RSV treatment could potentiate expression and differentiation markers like CD2, CD3, and CD8 and up-regulate the death receptor CD95/Fas at the surface of the target cells [24].

Resveratrol clinical trials in humans to investigate toxicity are very important together with the most up-to-date anti-cancer drugs to improve its therapeutic approaches, especially for treating lymphoma and leukemia.

## CONCLUSION

The locally purified resveratrol could serve as a multi-target drug that modulates the immune system to improve body defense in patients suffering from lymphoma and patients without lymphoma by differently altering cytokine levels in response to different resveratrol concentrations.

## Personal thanks

The authors are grateful to Biotechnology Center/Al-Nahrain University & National Center for Hematology/Al-Mustansiriyah University, Baghdad, Iraq, for their scientific support.
